# Editorial: Road traffic injury prevention and control

**DOI:** 10.3389/fpubh.2024.1549131

**Published:** 2025-01-07

**Authors:** Qingfeng Li, Guoqing Hu, Jaeyoung Jay Lee

**Affiliations:** ^1^Johns Hopkins University, Baltimore, MD, United States; ^2^Bloomberg School of Public Health, Johns Hopkins University, Baltimore, MD, United States; ^3^Central South University, Changsha, Hunan, China

**Keywords:** road traffic injuries (RTIs), injury prevention and control, system approach (SA), sustainable development goal (SDG), multisectorial collaboration

According to the World Health Organization (WHO), road traffic injuries (RTIs) cause ~1.19 million deaths globally each year. Over 90% of the fatalities on the roads occur in low- and middle-income countries (LMICs). More than half of these deaths are of vulnerable road users including pedestrians, cyclists, and motorcyclists. In addition to fatalities, between 20 to 50 million people sustain non-fatal injuries annually, many of which result in life-lasting disabilities. Given the persistent and devastating burden of road traffic crashes, there is an urgent need for further research and concerted action to address this critical public health issue. The articles in this Research Topic of Frontiers in Public Health tackle this pressing challenge, providing a comprehensive and multidimensional exploration of road traffic injury prevention and control.

This editorial synthesizes the contributions of the 11 articles in this Research Topic, which offer valuable evidence to inform future policy, practice, and research priorities in the field of road safety.

Several contributions examine RTI morbidity or mortality trends in broader contexts. Berheto et al. present a three-decade analysis of injury data in Ethiopia, highlighting a substantial decline in incidence and mortality but revealing inter-regional disparities that warrant localized prevention efforts. Their study underscores that, despite national progress, injuries remain a public health priority, requiring sustained and context-specific interventions. Meanwhile, Du et al. employ an age-period-cohort analysis in Jiangsu Province, China, documenting declining mortality rates for certain injuries, including those from road traffic crashes, yet noting a rise in unintentional falls. This intricate evolution underscores the importance of continuous surveillance to modify strategies as injury profiles shift over time.

Methodological advances improve our capacity to understand and prevent severe outcomes. Xiao et al. compare a rare events logistic regression model with a classic logit model to predict fatal crashes. Their findings demonstrate that rare event modeling more accurately identifies risk factors of fatal crash, providing a robust analytical tool for policymakers and researchers targeting high-risk conditions. Huang et al. adopts a Bayesian random-parameter spatial logistic model to investigate the effect of emergency medical service (EMS) response time on fatality risk in freeway crashes in China. Their work links shorter EMS response time with lower fatality odds, suggesting that improving pre-hospital emergency care capacity could be an effective life-saving strategy.

Environmental and contextual factors influencing RTIs are also highlighted. Li et al. focus on the effects of ambient temperature on traffic-related fatalities in Jinan, China. They find that both extremely high and low temperatures increase RTI risks, with delayed impacts across different modes of transportation. This research suggests the need to consider climate factors when planning road safety measures, especially as climate change intensifies temperature extremes. Similarly, Kim et al. investigate perceived pedestrian safety and show that while physical features like crosswalks and traffic signages, infrastructure quality, and comfort also shape subjective safety. Pedestrian-centered improvements must therefore consider not only engineering solutions but also user perception, fostering more friendly and secure walking environments.

The role of technology as a protective and preventive measure appears in multiple articles. Useche et al. systematically review the use of in-vehicle advanced driver assistance systems (ADAS) to prevent car-cyclist collisions. While studies support ADAS benefits, they also reveal potential downsides, such as driver overreliance. The studies also highlight a critical research gap: developing driver-training and awareness strategies to ensure ADAS complements, rather than substitutes for, attentive driving. Booker et al. discuss the Safe System Approach and how technology can help reduce serious RTIs. Their review points to the importance of evidence-based policy, context-specific technology adoption, and careful evaluation to achieve equitable safety gains worldwide.

Several articles delve into the behavioral, health system, and psychosocial dimensions of RTIs. Endalew et al. survey drivers of public transportation in Ethiopia, linking RTIs to a range of factors, from alcohol use to poor vehicle maintenance. These findings suggest that interventions cannot rely solely on infrastructure upgrades; rather, improved driver training, stricter enforcement, and better working conditions may be necessary to reduce crashes. Papadakaki et al. shine a spotlight on an often-overlooked consequence of RTIs: mental health impairment. Their review of European evidence shows that psychological repercussions can persist long after the initial trauma and that survivors often receive insufficient mental health support. This reveals a pressing need for integrated trauma care protocols that address both physical and psychological rehabilitation.

Focusing on survivors, Allen Ingabire et al. assess the quality of life of road traffic orthopedic injury victims in Rwanda two years after experiencing the injury. They find persistent functional and psychosocial limitations, emphasizing the value of rehabilitation and long-term support services. Beyond immediate trauma care, enhancing survivor reintegration and autonomy requires coordinated efforts, from policy implementation to health system strengthening. Similarly, while primarily focusing on trends, Du et al. also highlight the significance of targeted interventions that consider age and cohort patterns to protect at-risk populations, such as older adults, who sustain more severe injury outcomes.

These articles underscore the multifaceted nature of RTI prevention and control. Their collective insights demonstrate that achieving safer roads involves more than improved vehicles and infrastructure. It requires integrating robust epidemiological surveillance, advanced statistical methods, climate considerations, technology evaluation, driver training, equitable policies, and comprehensive rehabilitation. The Safe System Approach discussed by Booker et al. offers a valuable framework, but its success depends on interdisciplinary collaboration, political commitment, and sustainable resource allocation.

Future research should prioritize harmonizing data collection, establishing standardized metrics, and investing in longitudinal studies. Doing so will permit reasonable comparisons of interventions across regions, promoting knowledge transfer between different sociodemographic settings. Moreover, mental health outcomes and survivor quality of life need more attention, ensuring that post-crash care extends beyond EMSs and includes long-term psychosocial support.

In conclusion, the articles in this Research Topic collectively advance our knowledge of RTI epidemiology, risk factors, interventions, and outcomes. Policymakers, researchers, and practitioners need to collaborate to translate these insights into action. Achieving the Sustainable Development Goal of halving global road traffic deaths and injuries by 2030 requires coordinated efforts across sectors. As this Research Topic demonstrates, there is a wealth of evidence to guide these efforts and pave the way toward safer roads and healthier communities.

